# Pain and Its Management: Strategies and Outcomes in Older Adults With or at Risk for Knee Osteoarthritis

**DOI:** 10.1093/geroni/igaa057.659

**Published:** 2020-12-16

**Authors:** Alisa Johnson, Staja Booker, Josue Cardoso, Burel Goodin, Kimberly Sibille, Roger Fillingim

**Affiliations:** 1 University of Florida, Gainesville, Florida, United States; 2 The University of Florida, Gainesville, Florida, United States; 3 Pain Research and Intervention Center of Excellence, Gainesville, Florida, United States; 4 University of Alabama at Birmingham, Birmingham, Alabama, United States; 5 UNiversity of Florida, Gainesville, Florida, United States; 6 University of Florida, Pain Research and Intervention Center of Excellence, Gainesville, Florida, United States

## Abstract

Knee osteoarthritis (KOA) is a leading cause of mobility disability that is characterized by chronic pain among older adults. Non-Hispanic Blacks (NHBs) suffer disproportionately from non-Hispanic Whites (NHWs), reporting higher pain intensity and disability. It is unclear how these differences in symptomatology translate into different patterns of utilization for self-management (SM) of pain, and if such patterns are associated with underlying biological pain mechanisms. This multisite observational study examined (1) use of self-management strategies among older NHB and NHW adults with/at risk for KOA and (2) associations among self-management strategies, clinical and experimental pain. After approval from institutional IRBs, NHB and NHW older adults (N= 202) with knee pain completed the McGill Pain Questionnaire-Short Form, questions on treatment strategies (e.g., massage, ice, heat, medications), and quantitative sensory testing. Covariates included study site and education. On average, participants reported using 2 ± 1.65 SM strategies, with 79% endorsing at least one SM strategy. Analysis of covariance revealed that clinical pain differed by race/ethnicity and use of SM and/or medical treatments (p’s < 0.01). SM use did not differ by race/ethnicity, p = 0.15, but did differ significantly by gender, p < 0.05. Multiple linear regression demonstrated significant positive associations between SM and heat pain sensitivity for both NHBs and NHWs, (p < 0.05). SM is an important component of OA management for NHBs and NHWs. Our study is one of the first to show that SM use is significantly associated with pain mechanisms. Improved understanding will facilitate better mechanism-targeted pain management.

